# Perceptions of cannabis use risk to mental health among youth in Canada, England and the United States from 2017 to 2021

**DOI:** 10.1016/j.drugalcdep.2023.110904

**Published:** 2023-07-22

**Authors:** Maria K. Lemos, Eve Taylor, Elle Wadsworth, Jessica L. Reid, David Hammond, Katherine East

**Affiliations:** a National Addiction Centre, Institute of Psychiatry, Psychology and Neuroscience, King’s College London, London, UK; b RAND Europe, Cambridge, UK; c School of Public Health Sciences, Faculty of Health, University of Waterloo, Ontario, Canada

**Keywords:** Adolescents, Cannabinoids, Policy

## Abstract

**Background:**

There is little research examining perceptions of cannabis use risk to mental health in countries with differing cannabis regulations. This study therefore examines such perceptions among youth between 2017 and 2021 in Canada (non-medical cannabis legalized in October 2018), England (highly-restricted medical cannabis legalized November 2018), and the US (non-medical cannabis legal in some states).

**Methods:**

Seven repeat cross-sectional online surveys were conducted between July 2017 to August 2021 among youth aged 16–19 in Canada (N=29,420), England (N=28,155), and the US (N=32,974). Logistic regression models, stratified by country, were used to examine perceptions of cannabis use risk to mental health over time, adjusting for age group, sex, race/ethnicity, cannabis use and, for the US only, state-level cannabis legalization.

**Results:**

Perceptions that cannabis use posed “no risk” to mental health decreased between July 2017 and August 2021 in Canada (6.1–4.4%; AOR=0.64, 95% CI=0.52–0.78) and the US (14.0–11.3%; AOR=0.74, 0.65–0.84) but not England (3.7–4.5%; AOR=1.21, 0.97–1.52). No significant changes were observed from immediately before (August 2018) to after (August 2019) legalization of non-medical cannabis in Canada (AOR=0.99, 0.83–1.20) or highly-restricted medical cannabis in England (AOR=0.90, 0.70–1.17). In the US, perceptions of “no risk” were more likely in states where cannabis use was illegal (15.0%) compared with legal non-medical (12.2%) (AOR=0.68, 0.63–0.74).

**Conclusion:**

There were modest decreases in perceptions that cannabis use poses no risk to mental health in Canada and the US between 2017 and 2021 but no clear association with cannabis legalization status.

## 1. Introduction

Cannabis is the most commonly used psychoactive drug among youth worldwide, with approximately 5% of 15-to-16-year-olds reporting using cannabis at least once (World Health Organization, 2022). Prevalence also varies across countries; for example, in the US and Canada in 2019, approximately 18% of 16-to-19-year-olds reported using cannabis in the past 30 days, compared with 10% in England (Hammond et al., 2021).

Cannabis policies also vary across countries. In Canada, non-medical (or ‘recreational’) cannabis was legalized and regulated at the federal level in October 2018 for adults over 18 years old, although provinces vary in minimum legal age of sale and there are strict marketing controls and limits on personal possession and public use (Public First, 2021). In the US, as of November 2022, 19 states and the District of Columbia (DC) had legalized non-medical cannabis for adults over the age of 21 years, and 37 states had legalized medical cannabis. The US public continues to broadly favor cannabis legalization; for example, in 2022, 88% of US adults favored legalization of recreational cannabis and 59% favored legalization of medical cannabis (Pew Research Center, 2022). In England, non-medical cannabis remains illegal, but in 2018 the UK rescheduled some previously unlicensed cannabis-based products for prescription for specific medical conditions (UK Parliament, 2019); however, national guidelines were only released in November 2019 and prescriptions to date have been minimal (Freeman et al., 2019).

Cannabis use—particularly high-potency cannabis use—has been associated with mental ill-health, such as psychosis (Petrilli et al., 2022) and depression and anxiety among youth (Patton et al., 2002; Hines et al., 2020). Risk perceptions are likely to play an important role in cannabis use, including among youth susceptible to mental ill-health. Canadian research suggests that many youth believe cannabis can improve mental health and has few long-term health effects (McKiernan and Fleming, 2017). Indeed, despite the adverse effects on mental health, more than one-third of people who use cannabis in Canada and the US report doing so to manage mental health symptoms (Rup et al., 2022). Conversely, around 40% of youth in Canada and England, and 30% in the US, perceive cannabis use to be of great risk to mental health (Wadsworth and Hammond, 2019).

There is conflicting evidence on whether perceptions of cannabis risks change following legalization of medical and/or non-medical cannabis. As an increasing number of US states have legalized cannabis, youth perceptions that cannabis poses no risk to mental health have increased (Carliner et al., 2017). However, legalization in specific US states has not been associated with changes in youth perceptions of cannabis risks (Blevins et al., 2018). Specific components of legalization, including advertising and marketing of cannabis products, as well as public health campaigns that are implemented as part of legalization, may also impact risk perceptions. For example, in California (US), six months after cannabis legalization, exposure to cannabis advertising campaigns was associated with an increase in the perception that cannabis offers health benefits (D’Amico et al., 2018). In Canada, cannabis advertising is prohibited (Government of Canada, 2018) and, after federal legalization, public health campaigns were launched highlighting the risks of cannabis to mental health and how those who use cannabis can reduce their risks (Government of Canada, 2021).

The objective of this study was therefore to examine perceptions of risk of cannabis use to mental health among youth between 2017 and 2021 in three countries with differing cannabis legislation: Canada (non-medical cannabis legalized in October 2018), England (highly-restricted medical cannabis legalized in November 2018 but non-medical cannabis remains illegal), and the US (cannabis illegal federally, but non-medical cannabis was legal in 19 states and DC by 2022). This study extends work by Wadsworth and Hammond (2019), who examined cross-country differences in cannabis perceptions in 2017, to focus on perceptions before and after cannabis policy liberalization in the three countries.

## 2. Methods

### 2.1. Data source

The International Tobacco Control Policy Evaluation Project (ITC) Youth Tobacco and Vaping Survey is an online survey among youth in Canada, England, and the US. Repeat cross-sectional data were analyzed from 7 survey waves: annual waves conducted around August of each year (from 2017 to 2021), with additional semi-annual waves (around February) in 2020 and 2021. Respondents aged 16–19 years old were recruited from the Nielsen Consumer Insights Global Panel and their partner panels. This study was reviewed by and received ethical clearance from the University of Waterloo Research Ethics Board and the King’s College London Psychiatry, Nursing & Midwifery Research Ethics Subcommittee. See Technical Reports for further details (Hammond, 2022).

Of the n=91,694 survey respondents (Hammond, 2022), n=2536 were excluded from this study for refusing to answer measures of cannabis use, perceptions of cannabis use risk to mental health, US state, or ethnicity, leaving n=89,158 in this study’s analytic sample.

### 2.2. Measures

#### 2.2.1. Dependent variable: perceptions of risk to mental health

All respondents were asked “How much do you think people risk harming their MENTAL HEALTH when they use marijuana/cannabis on a regular basis?”, with response options (a) “No risk”, (b) “Slight risk”, (c) “Moderate risk”, (d) “Great risk” (e) “Don’t Know”, (f) “Refused”. Responses were recoded into: No risk (a) vs. Other (b-e) to reflect inaccurate vs. other perceptions. Refused (f) responses were excluded.

#### 2.2.2. Independent variables

Key predictors were survey wave (July 2017, August 2018, August 2019, February 2020, August 2020, February 2021, August 2021) and, for the US only, legal status of cannabis (illegal, legal medical, legal non-medical; derived based on state, survey wave, and policy implementation date; see Table S1, Supplementary Materials). Covariates were sex (male, female), age group (16–17, 18–19), race/ethnicity (other/mixed, White only; based on country-specific questions), and cannabis use (never used; ever used, but not in the past 30 days; used in the past 30 days). Regression models were stratified by country of residence (Canada, England, US).

### 2.3. Analysis

Analyses were conducted using SPSS (IBM), version 26.

Post-stratification sample weights were constructed for each country based on sex-by-age-by-region in Canada and England and sex-by-age-by-region-by-race/ethnicity in the US, and calibrated to wave 1 proportions for student status (student vs. not) and school grades (<70%, don’t know, and refused; 70–79%; 80–89%; 90–100%) for subsequent waves, as well as the past 30-day cigarette smoking prevalence trend observed in national surveys in Canada and the US. Weights were rescaled to each country’s sample size. See Technical Reports for further details (Hammond, 2022).

The number and proportion of participants reporting that cannabis use posed ‘no risk’ to mental health were reported by country and survey wave. Then, three separate binary logistic regression models were estimated (one per country), adjusting for age group, sex, race/ethnicity, cannabis use and, in the US only, state-level cannabis legalization status. A sensitivity analysis was also run without adjusting for cannabis use, but this did not change the interpretation of the findings. Wave-by-wave comparisons were then subsequently reported to examine whether perceptions of risk to mental health changed each wave compared to the prior wave, with a particular focus on immediately prior (August 2018) to after (August 2019) legalization status changed in Canada and England.

## 3. Results

The majority of the sample were aged 18–19 years (50.6–53.5%, depending on country), male (51.0–51.2%), identified as White (56.2–57.8%), and had never used cannabis (69.4–76.6%) (Table 1; characteristics by country and wave are in Table S2).

**Fig. 1.**
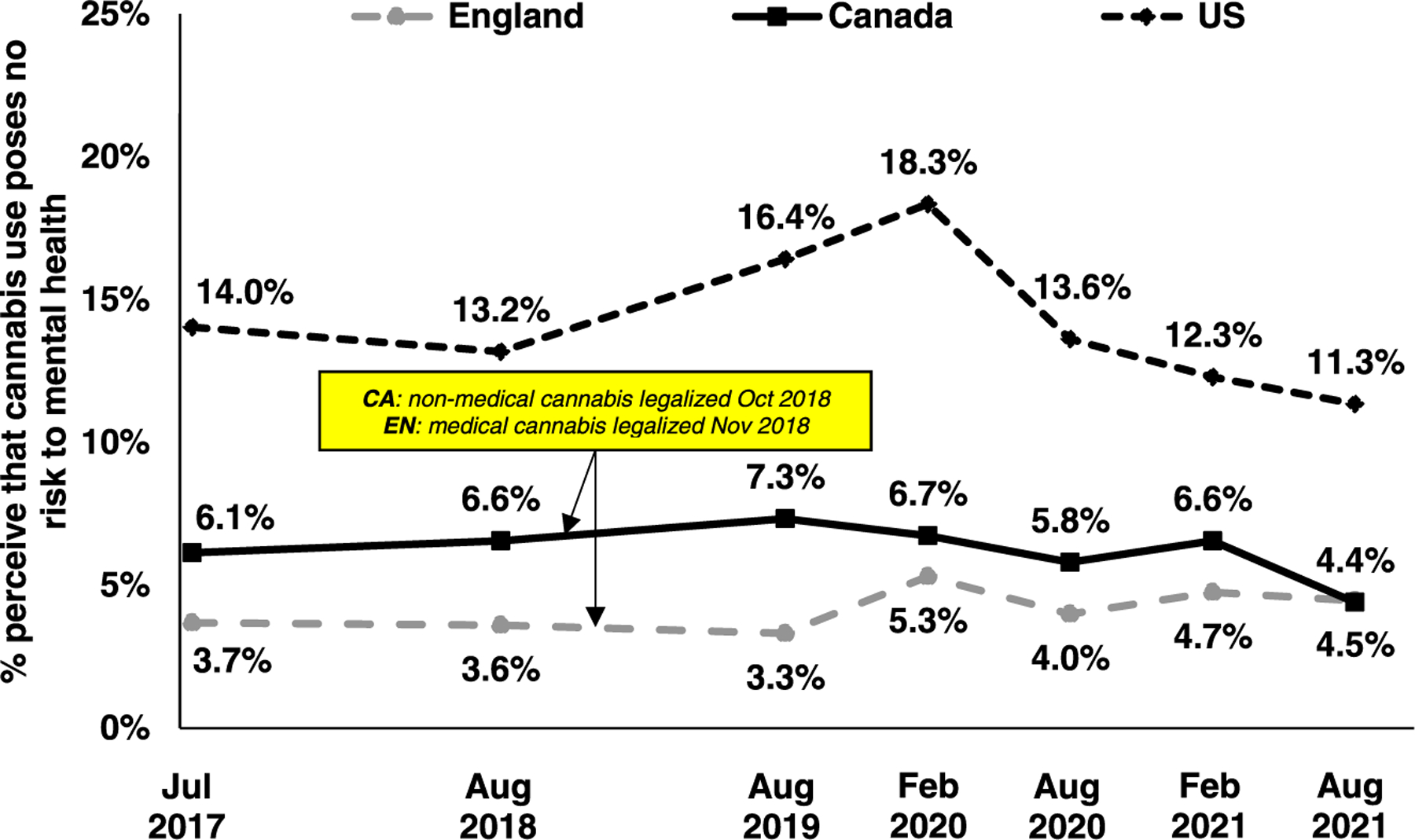
Perception that cannabis use poses no risk to mental health among youth in Canada (N=28,581), England (N=27,856) and the US (N=32,721) from 2017 to 2021. Table S4 shows the full range of responses.

Fig. 1 shows perceptions of cannabis use risk to mental health from 2017 to 2021 in each country. Table 1 shows the results of the logistic regression analyses. Overall, few youth perceived that cannabis posed “no risk” to mental health (ranging from 3.3% and 18.3%, depending on the country and survey wave). In Canada, there was little change in perceptions that cannabis use posed “no risk” to mental health during the study period except for a decrease in August 2021 (4.4%, vs. 6.1% in July 2017, AOR=0.64, 95% CI=0.52–0.78). In England, perceptions that cannabis posed “no risk” to mental health increased significantly from July 2017 to February 2020 (3.7–5.3%, AOR=1.31, 1.05–1.63) but then remained relatively stable. In the US, perceptions that cannabis use posed “no risk” to mental health increased from 14.0% in July 2017–18.3% in February 2020 (AOR=1.16, 1.03–1.31) before decreasing to 11.3% in August 2021, below 2017 levels (AOR=0.74, 0.65–0.84).

In Canada and England, no significant changes in perceptions that cannabis use posed “no risk” to mental health were observed from immediately before (August 2018) to after (August 2019) legalization of non-medical cannabis in Canada (6.6–7.3%, AOR=0.99, 0.83–1.20) or legalization of medical cannabis in England (3.6–3.3%, AOR=0.90, 0.70–1.17) (Table S3). The most notable change was observed between August 2019 and February 2020, but effects were not consistent across countries: perceptions of no risk decreased in Canada (AOR=0.83, 0.70–0.99) but increased in England (AOR=1.49, 1.18–1.89) and the US (AOR=1.15. 1.02–1.30) (Table S3).

In the US, the proportion of youth reporting ‘no risk’ of cannabis harm to mental health was significantly higher in states where cannabis was illegal (15.0%) compared with states that had legalized non-medical cannabis (12.2%) (Table 1). In all three countries, the proportion reporting that cannabis use posed “no risk” to mental health was higher among those who used cannabis in the past 30 days (CA=19.1%, EN=14.7%, US=38.2%), than those who had never used cannabis (CA=2.9%, EN=2.4%, US=7.6%) (Table 1).

## 4. Discussion

Perceptions of risk of cannabis use to mental health fluctuated over the study period in all three countries, with an overall modest decrease in perceptions that cannabis use posed no risk to mental health in Canada and the US. The most pronounced increases in this belief were observed in the US and England between August 2019 and February 2020, but these were not sustained through to August 2021.

Consistent with previous research (Blevins et al., 2018), there was little evidence to suggest that perceptions of cannabis use risk to mental health changed from immediately before to after legalization of non-medical cannabis in Canada in October 2018, or of highly-restricted medical cannabis in England in November 2018. Moreover, consistent with previous publications using 2017 ITC Youth survey data (Wadsworth and Hammond, 2019), more youth from the US perceived that cannabis use posed no risk to mental health than youth in Canada and England, and such differences continued after Canada legalized non-medical cannabis and despite cannabis remaining illegal in many US states. Prevalence of perceptions that cannabis use posed no risk to mental health decreased overall during the study period in the US despite an increasing number of states legalizing either medical or non-medical cannabis, and youth in US states where cannabis was illegal were more likely to report ‘no risk’, contrary to prior research (Carliner et al., 2017) and the idea that legalization reduces perceptions of cannabis risks. Overall, our findings suggest that legalization does not appear to have an immediate or substantial impact on reducing risk perceptions of cannabis use.

In Canada and England, any legalization effects may have been diminished by public/media discussions in the period leading up to legalization. For example, in 2017, there was wide-spread media coverage of a campaign in England to prescribe medical cannabis for a child with epilepsy (Deacon, 2019) and the federal bill to legalize cannabis in Canada was also widely reported on when it was first passed (Gunning and Illes, 2021); this publicity may have decreased perceptions of cannabis risks. Further, despite medical legalization of cannabis in England, access is heavily restricted and very few people have received a prescription (Dowden, 2021) and so legalization may not have made any noticeable difference to perceptions. Future studies should examine legalization effects over a longer period of time; risk perceptions are likely to change gradually with greater exposure to retail and other sources of promotion, particularly among new cohorts of youth as they age into the period of cannabis initiation.

An unexpected finding that does not clearly correspond with policy changes was the increase observed in the perception that cannabis poses no risk to mental health in the US and England between August 2019 and February 2020. The change in England may be a lagged effect of medical legalization on risk perceptions; however, this change was not sustained, as the proportion reporting ‘no risk’ decreased in August 2020. Observed changes between August 2019 and February 2020 also coincided with the outbreak of lung injuries in September 2019 that was primarily attributable to vaping THC products contaminated with Vitamin E Acetate in the US (Centers for Disease and Control Prevention, 2021), although effects would have been expected in the opposite direction (i. e., increased perceptions of harm) and also in Canada, which was also affected by the outbreak, albeit to a lesser extent than the US. The beginning of the COVID-19 pandemic may also have impacted cannabis use and perceptions of risk (Layman et al., 2022), but again we would have expected effects to be similar across the three countries.

Limitations of this study include that it was cross-sectional, so it was not possible to draw inferences from within-person changes in perceptions of risks of cannabis use over time. We were also limited to seven time points approximately six- or 12-months apart, with confounding present (e.g., COVID-19). More granular data with more time points are required to increase confidence in cannabis policy effects. However, large samples of youth were surveyed, and data were weighted to enhance representativeness.

## 5. Conclusion

There were modest decreases in perceptions that cannabis use poses no risk to mental health among youth in Canada and the US between 2017 and 2021, but no clear association with cannabis legalization status. Perceptions remained stable between 2017 and 2021 in England. Any effects of legalization must be examined over a longer period of time, considering changing cannabis laws as well as prevalence, attitudes, and exposure to promotions and education campaigns.

## Supplementary Material

Supplementaty Materials

## Figures and Tables

**Table 1:** Adjusted logistic regression models of the perception that cannabis use poses no risk to mental health and survey wave, age, sex, race/ethnicity, past 30-day cannabis use, and legal status, stratified by country.

	Canada (N=28,581)				England (N=27,856)				US (N=32,721)			
	N (%) sample	N (%) perceive ‘no risk’	AOR (95% CI)^1^	p	N (%) sample	N (%) perceive ‘no risk’	AOR (95% CI)^1^	p	N (%) sample	N (%) perceive ‘no risk’	AOR (95% CI)^2^	p
**Survey wave**																								
1 (July/Aug 2017)	3997 (13.9)	240 (6.1)	1	**ref**	3939 (14.1)	135 (3.7)	1	**ref**	4033 (12.3)	583 (14.0)	1	**ref**
2 (Aug/Sept 2018)	3516 (12.4)	259 (6.6)	1.10 (0.90–1.33)	0.352	3800 (13.6)	137 (3.6)	0.97 (0.76–1.24)	0.830	4001 (12.2)	542 (13.2)	0.90 (0.79–1.03)	0.116
3 (Aug/Sept 2019)	3981 (13.9)	338 (7.3)	1.09 (0.91–1.31)	0.333	3396 (12.2)	123 (3.3)	0.88 (0.68–1.13)	0.323	3908 (12.0)	741 (16.4)	1.01 (0.88–1.15)	0.918
3.5 (Feb/Mar 2020)	4064 (14.2)	324 (6.7)	0.91 (0.76–1.09)	0.312	4154 (14.9)	247 (5.3)	**1.31 (1.05–1.63)**	**0.016**	5044 (15.4)	925 (18.3)	**1.16 (1.03–1.31)**	**0.017**
4 (Aug 2020)	4130 (14.4)	247 (5.8)	0.88 (0.73–1.06)	0.187	4194 (15.1)	176 (4.0)	1.05 (0.84–1.33)	0.666	5860 (18.0)	799 (13.6)	0.89 (0.79–1.00)	0.057
4.5 (Feb/Mar 2021)	4446 (15.6)	286 (6.6)	0.95 (0.79–1.13)	0.545	4169 (15.0)	192 (4.7)	1.24 (0.99–1.55)	0.057	5128 (15.7)	644 (12.3)	**0.78 (0.68–0.88)**	**<0.001**
5 (Aug/Sept2021)	4447 (15.5)	191 (4.4)	**0.64 (0.52–0.78)**	**<0.001**	4204 (15.1)	187 (4.5)	1.21 (0.97–1.52)	0.094	4747 (14.5)	545 (11.3)	**0.74 (0.65–0.84)**	**<0.001**
**Age group**																								
16–17 years	12,531 (47.7)	763 (5.6)	1	ref	11,547 (49.1)	465 (3.4)	1	ref	15,629 (49.4) 2014 (12.4)	1	ref	
18–19 years	16,050 (52.3)	1010 (6.7)	0.94 (0.85–1.05)	0.269	16,309 (50.9)	706 (5.0)	**1.20 (1.06–1.36)**	**0.003**	17,092 (50.6)	2629 (15.8)	0.99 (0.93–1.06)	0.761
Sex												
Male	10,676 (51.0)	828 (7.2)	1	Ref	10,056 (51.2)	544 (5.1)	1	ref	10,219 (51.0)	1556 (14.6)	1	ref
Female	17,905 (49.0)	1057 (5.2)	**0.69 (0.63–0.77)**	**<0.001**	17,800 (48.8)	653 (3.3)	**0.65 (0.58–0.74)**	**<0.001**	22,502 (49.0)	**3223 (13.7)**	**0.85 (0.80–0.91)**	**<0.001**
**Race/ethnicity**																								
Other/Mixed	12,491 (43.8)	704 (5.1)	1	ref	7132 (24.2)	272 (3.7)	1	ref	14,631 (28.4)	1928 (13.0)	1	ref
White (only)	16,090 (56.2)	1181 (7.0)	**1.17 (1.06–1.30)**	**0.002**	20,724 (75.8)	925 (4.4)	1.13 (0.98–1.31)	0.101	18,090 (71.6)	2851 (14.6)	**1.17 (1.08–1.26)**	**<0.001**
**Cannabis Use**																								
Never	18,866 (69.4)	582 (2.9)	1	ref	20,780 (76.6)	506 (2.4)	1	ref	21,630 (70.0)	1741 (7.6)	1	ref
Ever	4464 (14.5)	310 (7.5)	**2.68 (2.32–3.10)**	**<0.001**	4095 (13.5)	258 (6.9)	**2.98 (2.55–3.48)**	**<0.001**	5149 (14.3)	938 (19.9)	**3.12 (2.86–3.41)**	**<0.001**
Past 30-day	5251 (16.1)	881 (19.1)	**7.88 (7.04–8.82)**	**<0.001**	2981 (9.9)	407 (14.7)	**6.71 (5.84–7.71)**	**<0.001**	5942 (15.7)	1965 (38.2)	**7.82 (7.24–8.44)**	**<0.001**
**Legal status (US only)**												
Illegal	-		-	-	-	-	-	-	9821 (33.5)	1533 (15.0)	1	ref
Legal medical	-		-	-	-	-	-	-	13,941 (37.8)	2136 (14.9)	**0.91 (0.85–0.99)**	**0.019**
Legal non-medical	-		-	-	-	-	-	-	8959 (28.7)	1110 (12.2)	**0.68 (0.63–0.74)**	**<0.001**

Bolded values represent significance at p<0.05. Three separate regressions were run for Canada, England and the US. All data except N are weighted.

1 Adjusting for survey wave, age group, sex, race/ethnicity, cannabis use.

2 Adjusting for survey wave, age group, sex, race/ethnicity, cannabis use, and state-level cannabis legalization status.
