# Supplementary material for: Perceptions of cannabis use risk to mental health among youth in Canada, England and the United States from 2017 to 2021
Source: Drug Alcohol Depend. Author manuscript; Available in PMC 2024 Feb 7. (PMC10847957; doi:10.1016/j.drugalcdep.2023.110904)
Supplement: Supplementaty Materials [file NIHMS1958150-supplement-Supplementaty_Materials.docx]

**Table S1. Cannabis laws in the United States by state over time, from July 2017 to August 2021. Coded as; 0=Illegal, 1=Legal Medicinal, 2=Legal Non-Medicinal. All dates are implementation dates.**

| STATE | Medical legal | Non-medical legal | W1 (Aug 2017) | W2 (Aug 2018) | W3 (Aug 2019) | W3.5 (Feb 2020) | W4 (Aug 2020) | W4.5 (Feb 2021) | W5 (Aug 2021) |
| --- | --- | --- | --- | --- | --- | --- | --- | --- | --- |
| Alabama | 2021 (May) | N/A | 0 | 0 | 0 | 0 | 0 | 0 | 1 |
| Alaska | 1998 | 2015 (Feb) | 2 | 2 | 2 | 2 | 2 | 2 | 2 |
| Arizona | 2010 | 2020 (Nov) | 1 | 1 | 1 | 1 | 1 | 2 | 2 |
| Arkansas | 2016 | N/A | 1 | 1 | 1 | 1 | 1 | 1 | 1 |
| California | 1996 | 2016 (Nov) | 2 | 2 | 2 | 2 | 2 | 2 | 2 |
| Colorado | 2001 | 2012 (Dec) | 2 | 2 | 2 | 2 | 2 | 2 | 2 |
| Connecticut | 2012 | 2021 (Jul) | 1 | 1 | 1 | 1 | 1 | 1 | 2 |
| Delaware | 2011 | N/A | 1 | 1 | 1 | 1 | 1 | 1 | 1 |
| DC | 2010 | 2015 (Feb) | 2 | 2 | 2 | 2 | 2 | 2 | 2 |
| Florida | 2016 | N/A | 1 | 1 | 1 | 1 | 1 | 1 | 1 |
| Georgia | N/A | N/A | 0 | 0 | 0 | 0 | 0 | 0 | 0 |
| Hawaii | 2000 | N/A | 1 | 1 | 1 | 1 | 1 | 1 | 1 |
| Idaho | N/A | N/A | 0 | 0 | 0 | 0 | 0 | 0 | 0 |
| Illinois | 2013 | 2020 (Jan) | 1 | 1 | 1 | 2 | 2 | 2 | 2 |
| Indiana | N/A | N/A | 0 | 0 | 0 | 0 | 0 | 0 | 0 |
| Iowa | N/A | N/A | 0 | 0 | 0 | 0 | 0 | 0 | 0 |
| Kansas | N/A | N/A | 0 | 0 | 0 | 0 | 0 | 0 | 0 |
| Kentucky | N/A | N/A | 0 | 0 | 0 | 0 | 0 | 0 | 0 |
| Louisiana | 2016 | N/A | 1 | 1 | 1 | 1 | 1 | 1 | 1 |
| Maine | 1999 | 2017 (Jan) | 2 | 2 | 2 | 2 | 2 | 2 | 2 |
| Maryland | 2014 | N/A | 1 | 1 | 1 | 1 | 1 | 1 | 1 |
| Massachusetts | 2013 | 2016 (Dec) | 2 | 2 | 2 | 2 | 2 | 2 | 2 |
| Michigan | 2008 | 2018 (Dec) | 1 | 1 | 2 | 2 | 2 | 2 | 2 |
| Minnesota | 2014 | N/A | 1 | 1 | 1 | 1 | 1 | 1 | 1 |
| Mississippi | N/A | N/A | 0 | 0 | 0 | 0 | 0 | 0 | 0 |
| Missouri | 2018 (Dec) | N/A | 0 | 0 | 1 | 1 | 1 | 1 | 1 |
| Montana | 2004 | 2021 (Jan) | 1 | 1 | 1 | 1 | 1 | 2 | 2 |
| Nebraska | N/A | N/A | 0 | 0 | 0 | 0 | 0 | 0 | 0 |
| Nevada | 2001 | 2017 (Jan) | 2 | 2 | 2 | 2 | 2 | 2 | 2 |
| New Hampshire | 2013 | N/A | 1 | 1 | 1 | 1 | 1 | 1 | 1 |
| New Jersey | 2010 | 2021 (Jan) | 1 | 1 | 1 | 1 | 1 | 2 | 2 |
| New Mexico | 2007 | 2021 (Jun) | 1 | 1 | 1 | 1 | 1 | 1 | 2 |
| New York | 2014 | 2021 (Mar) | 1 | 1 | 1 | 1 | 1 | 1 | 2 |
| North Carolina | N/A | N/A | 0 | 0 | 0 | 0 | 0 | 0 | 0 |
| North Dakota | 2016 | N/A | 1 | 1 | 1 | 1 | 1 | 1 | 1 |
| Ohio | 2016 | N/A | 1 | 1 | 1 | 1 | 1 | 1 | 1 |
| Oklahoma | 2018 (June) | N/A | 0 | 1 | 1 | 1 | 1 | 1 | 1 |
| Oregon | 1998 | 2015 (Jul) | 2 | 2 | 2 | 2 | 2 | 2 | 2 |
| Pennsylvania | 2016 | N/A | 1 | 1 | 1 | 1 | 1 | 1 | 1 |
| Rhode Island | 2006 | N/A | 1 | 1 | 1 | 1 | 1 | 1 | 1 |
| South Carolina | N/A | N/A | 0 | 0 | 0 | 0 | 0 | 0 | 0 |
| South Dakota | 2021 (Aug) | N/A | 0 | 0 | 0 | 0 | 0 | 0 | 1 |
| Tennessee | N/A | N/A | 0 | 0 | 0 | 0 | 0 | 0 | 0 |
| Texas | N/A | N/A | 0 | 0 | 0 | 0 | 0 | 0 | 0 |
| Utah | 2018 (Dec) | N/A | 0 | 0 | 1 | 1 | 1 | 1 | 1 |
| Vermont | 2004 | 2018 (Jul) | 1 | 2 | 2 | 2 | 2 | 2 | 2 |
| Virginia | 2020 (Jul) | 2021 (Jul) | 0 | 0 | 0 | 0 | 1 | 1 | 2 |
| Washington | 1998 | 2012 (Dec) | 2 | 2 | 2 | 2 | 2 | 2 | 2 |
| West Virginia | 2019 (Jul) | N/A | 0 | 0 | 1 | 1 | 1 | 1 | 1 |
| Wisconsin | N/A | N/A | 0 | 0 | 0 | 0 | 0 | 0 | 0 |
| Wyoming | N/A | N/A | 0 | 0 | 0 | 0 | 0 | 0 | 0 |

**Sources:**

National Conference of State Legislatures: https://www.ncsl.org/research/health/state-medical-marijuana-laws.aspx

ProCon: https://medicalmarijuana.procon.org/legal-medical-marijuana-states-and-dc/ and

https://marijuana.procon.org/legal-recreational-marijuana-states-and-dc/

NORML: https://norml.org/laws/

**Table S2. Sample characteristics (unweighted n, weighted %) by each country and survey wave (N=89,158).**

|  |  |  | July 2017 | August 2018 | August 2019 | February 2020 | August 2020 | February 2021 | August 2021 |
| --- | --- | --- | --- | --- | --- | --- | --- | --- | --- |
| Country | **Demographic Information** | | **N(%)** | **N(%)** | **N(%)** | **N(%)** | **N(%)** | **N(%)** | **N(%)** |
| Canada (N=28,581) | **Age group** | **16-17 years** | 1569 (47.9) | 1318 (46.9) | 1722 (47.4) | 1670 (47.5) | 2113 (47.7) | 2131 (47.4) | 2008 (48.9) |
|  |  | **18-19 years** | 2428 (52.1) | 2198 (53.1) | 2259 (52.6) | 2394 (52.5) | 2017 (52.3) | 2315 (52.6) | 2439 (51.1) |
|  | **Sex** | **Male** | 1389 (51.2) | 1591 (50.4) | 1480 (51.0) | 1543 (51) | 1655 (51.1) | 1490 (51.3) | 1528 (50.9) |
|  |  | **Female** | 2608 (48.8) | 1925 (49.6) | 2501 (49.0) | 2521 (49) | 2475 (48.9) | 2956 (48.7) | 2919 (49.1) |
|  | **Race/ ethnicity** | **Other/Mixed** | 1847 (41.6) | 1713 (49.2) | 1743 (44.3) | 1743 (43.0) | 1718 (42.4) | 1725 (40.3) | 2002 (46.4) |
|  |  | **White (only)** | 2150 (58.4) | 1803 (50.8) | 2238 (55.7) | 2321 (57.0) | 2412 (57.6) | 2721 (59.7) | 2445 (53.6) |
| England (N=27,856) | **Age group** | **16-17 years** | 1586 (48.7) | 1148 (48.4) | 1486 (48.8) | 1831 (49.1) | 1642 (49.3) | 2017 (49.1) | 1837 (50.2) |
|  |  | **18-19 years** | 2353 (51.3) | 2652 (51.6) | 1910 (51.2) | 2323 (50.9) | 2552 (50.7) | 2152 (50.9) | 2367 (49.8) |
|  | **Sex** | **Male** | 1672 (51.2) | 1363 (51.1) | 1195 (51.1) | 1555 (51.2) | 1530 (51.4) | 1299 (51.4) | 1442 (51.2) |
|  |  | **Female** | 2267 (48.8) | 2437 (48.9) | 2201 (48.9) | 2599 (48.8) | 2664 (48.6) | 2870 (48.6) | 2762 (48.8) |
|  | **Race /ethnicity** | **Other/Mixed** | 849 (20.6) | 923 (21.9) | 807 (23.0) | 916 (21.6) | 1097 (24.5) | 1198 (27.5) | 1342 (29.5) |
|  |  | **White (only)** | 3090 (79.4) | 2877 (78.1) | 2589 (77.0) | 3238 (78.4) | 3097 (75.5) | 2971 (72.5) | 2862 (70.5) |
| US (N=32,721) | **Age group** | **16-17 years** | 1775 (50.2) | 2031 (49.5) | 1649 (49.3) | 2401 (49.2) | 2983 (49.1) | 2861 (49.2) | 1929 (49.3) |
|  |  | **18-19 years** | 2258 (49.8) | 1970 (50.5) | 2259 (50.7) | 2643 (50.8) | 2877 (50.9) | 2267 (50.8) | 2818 (50.7) |
|  | **Sex** | **Male** | 1595 (51.1) | 1425 (50.9) | 1207 (51.1) | 1668 (51.0) | 1721 (51.1) | 1417 (50.8) | 1186 (50.8) |
|  |  | **Female** | 2438 (48.9) | 2576 (49.1) | 2701 (48.9) | 3376 (49.0) | 4139 (48.9) | 3711 (49.2) | 3561 (49.2) |
|  | **Race/ ethnicity** | **Other/Mixed** | 1393 (26.6) | 1528 (26.5) | 1754 (25.9) | 1958 (26.4) | 2943 (29.3) | 2509 (32.6) | 2546 (29.9) |
|  |  | **White (only)** | 2640 (73.4) | 2473 (73.5) | 2154 (74.1) | 3086 (73.6) | 2917 (70.7) | 2619 (67.4) | 2201 (70.1) |

**Table S3. Wave-by-wave comparisons of the perception that cannabis use poses no risk to mental health, stratified by country and adjusting for covariates (N=89,158).**

|  | Canada (N=28,581) ^1^ | | England (N=27,856) ^1^ | | US (N=32,721) ^2^ | | |
| --- | --- | --- | --- | --- | --- | --- | --- |
|  | **AOR (95% CI)** | **p** | **AOR (95% CI)** | **p** | **AOR (95% CI)** | **p** | |
| August 2018 vs. July 2017 | 1.10 (0.90-1.33) | .352 | 0.97 (0.76-1.24) | .830 | 0.90 (0.79-1.03) | .116 | |
| August 2019 vs. August 2018 | 0.99 (0.83-1.20) | .991 | 0.90 (0.70-1.17) | .438 | 1.11 (0.97-1.27) | .116 | |
| February 2020 vs. August 2019 | **0.83 (0.70-0.99)** | **.040** | **1.49 (1.18-1.89)** | **<.001** | **1.15 (1.02-1.30)** | **.020** | |
| August 2020 vs. February 2020 | 0.97 (0.81-1.17) | .732 | **0.80 (0.65-0.99)** | **.041** | **0.77 (0.69-0.86)** | **<.001** | |
| February 2021 vs. August 2020 | 1.07 (0.90-1.29) | .441 | 1.18 (0.95-1.46) | .131 | **0.87 (0.78-0.98)** | **.024** | |
| August 2021 vs. February 2021 | **0.67 (0.56-0.82)** | **<.001** | 0.98 (0.79-1.20) | .823 | 0.95 (0.84-1.08) | .465 | |
| Bolded values represent significance at p<0.05. Three separate regressions were run for Canada, England and the US. All data except N are weighted.  ^1^ Adjusting for age group, sex, race/ethnicity, cannabis use.  ^2^ Adjusting for age group, sex, race/ethnicity, cannabis use, and state-level cannabis legalization status. | | | | | | |  |

**Table S4. Weighted % (unweighted N) of perceptions of the risks of cannabis use risk to mental health by each country and survey wave (N=89,158).**

|  |  | July 2017 | | August 2018 | August 2019 | February 2020 | August 2020 | February 2021 | August 2021 |
| --- | --- | --- | --- | --- | --- | --- | --- | --- | --- |
| Country | **Perception** | **N(%)** | **N(%)** | | **N(%)** | **N(%)** | **N(%)** | **N(%)** | **N(%)** |
| Canada (N=28,581) | **No risk** | 240 (6.1) | | 259 (6.6) | 338 (7.3) | 324 (6.7) | 247 (5.8) | 286 (6.6) | 191 (4.4) |
|  | **Slight risk** | 899 (21.9) | | 749 (20) | 826 (19.5) | 977 (21.8) | 789 (19.1) | 813 (18.7) | 806 (18.5) |
|  | **Moderate risk** | 1119 (27.4) | | 1028 (29.5) | 1113 (27.5) | 1167 (28.1) | 1162 (27.9) | 1304 (29) | 1275 (28.1) |
|  | **Great risk** | 1519 (39.1) | | 1238 (37.4) | 1437 (39) | 1297 (35.8) | 1568 (38.7) | 1666 (36.9) | 1796 (40.6) |
|  | **Don’t know** | 220 (5.4) | | 242 (6.6) | 267 (6.7) | 299 (7.5) | 364 (8.6) | 377 (8.8) | 379 (8.4) |
| England (N=27,856) | **No risk** | 135 (3.7) | | 137 (3.6) | 123 (3.3) | 247 (5.3) | 176 (4.0) | 192 (4.7) | 187 (4.5) |
|  | **Slight risk** | 797 (20.0) | | 766 (19.4) | 633 (18.4) | 816 (19.2) | 822 (18.8) | 724 (17.2) | 741 (16.9) |
|  | **Moderate risk** | 1113 (28) | | 1107 (28.2) | 970 (28.8) | 1153 (27.9) | 1148 (26.9) | 1174 (27.6) | 1181 (27.3) |
|  | **Great risk** | 1723 (43.8) | | 1571 (41.9) | 1479 (43.9) | 1649 (41) | 1743 (42.8) | 1768 (42.7) | 1775 (43.2) |
|  | **Don’t know** | 171 (4.5) | | 219 (6.9) | 191 (5.6) | 289 (6.7) | 305 (7.4) | 311 (7.7) | 320 (8.2) |
| US (N=32,721) | **No risk** | 583 (14.0) | | 542 (13.2) | 741 (16.4) | 925 (18.3) | 799 (13.6) | 644 (12.3) | 545 (11.3) |
|  | **Slight risk** | 986 (24.1) | | 821 (19.5) | 904 (22.3) | 1223 (22.5) | 1324 (23.8) | 1078 (21.8) | 1081 (21.5) |
|  | **Moderate risk** | 978 (24.1) | | 981 (24.6) | 880 (23.2) | 1127 (22.6) | 1489 (25.2) | 1293 (25.4) | 1230 (26.3) |
|  | **Great risk** | 1216 (31.2) | | 1269 (32.7) | 1045 (29.5) | 1254 (26.2) | 1720 (28.1) | 1612 (31.1) | 1449 (31.7) |
|  | **Don’t know** | 270 (6.6) | | 388 (10.1) | 338 (8.6) | 515 (10.4) | 528 (9.2) | 501 (9.3) | 442 (9.1) |
